# Tracing diagnosis trajectories over millions of patients reveal an unexpected risk in schizophrenia

**DOI:** 10.1038/s41597-019-0220-5

**Published:** 2019-10-15

**Authors:** Hyojung Paik, Matthew J. Kan, Nadav Rappoport, Dexter Hadley, Marina Sirota, Bin Chen, Udi Manber, Seong Beom Cho, Atul J. Butte

**Affiliations:** 1 Bakar Computational Health Sciences Institute, University of California, San Francisco, 550 16th Street, San Francisco, CA 9414 USA; 2 Department of Pediatrics, University of California, San Francisco, 550 16th Street, San Francisco, CA 94143 USA; 3 Korea Institute of Science and Technology Information, Center for Supercomputing Application, Division of Supercomputing, Daejeon, 34141 South Korea; 4 National Institute of Health, Division of Bio-Medical Informatics, Center for Genome Science, OHTAC, 187 Osongsaengmyeong2(i)-ro, Gangoe-myeon, Cheongwon-gun, ChoongchungBuk-do South Korea; 5 Department of Medicine, University of California, San Francisco, 505 Parnassus Avenue, San Francisco, CA 94143 USA

**Keywords:** Diagnosis, Preclinical research, Schizophrenia, Risk factors

## Abstract

The identification of novel disease associations using big-data for patient care has had limited success. In this study, we created a longitudinal disease network of traced readmissions (disease trajectories), merging data from over 10.4 million inpatients through the Healthcare Cost and Utilization Project, which allowed the representation of disease progression mapping over 300 diseases. From these disease trajectories, we discovered an interesting association between schizophrenia and rhabdomyolysis, a rare muscle disease (incidence < 1E-04) (relative risk, 2.21 [1.80–2.71, confidence interval = 0.95], *P*-value 9.54E-15). We validated this association by using independent electronic medical records from over 830,000 patients at the University of California, San Francisco (UCSF) medical center. A case review of 29 rhabdomyolysis incidents in schizophrenia patients at UCSF demonstrated that 62% are idiopathic, without the use of any drug known to lead to this adverse event, suggesting a warning to physicians to watch for this unexpected risk of schizophrenia. Large-scale analysis of disease trajectories can help physicians understand potential sequential events in their patients.

## Introduction

One of the most basic aspects of clinical care in hospitals is understanding disease and mortality risk factors, particularly for rare but preventable diseases or outcomes^1–3^. Although mapping disease relationships has a long history, the recent advent of digitalized health records and disease registries has led to an enhanced ability to organize and analyze healthcare data. The availability of these data allows the recapitulation of known temporal disease correlations using nonlongitudinal data^4^, exploration of unordered disease pairs^5,6^, and enables network analyses of disease relationships at a national scale^7^. However, while big-data analytics have aided the ability to visualize, search, and organize health data^8,9^, there has been limited success in identifying and validating novel temporal relationships between diseases that would meaningfully change clinical care.

Here, we showcase a novel temporal disease association that was discovered in the course of a large-scale analysis of California inpatient hospitalizations using data from the Healthcare Cost and Utilization Project (HCUP)^10^. By using the California State Inpatient Database (CA SID) from HCUP, we analyzed longitudinal inpatient discharge data collected between 1980 and 2010 from 350 California hospitals, representing 10.4 million ethnically diverse individuals. Based on inpatient diagnoses, we identified temporal correlations between disease pairs^7^ and concatenated these correlations to create a network-based representation of disease trajectories from initial hospitalization until death in a hospital. By using a combination of computational analysis and healthcare expert curation, an unexpected relationship between inpatient admissions for schizophrenia, a psychiatric disorder, and readmission for rhabdomyolysis was discovered.

Rhabdomyolysis is a rare disease of muscle breakdown that can lead to severe outcomes for patients, including kidney failure^11^. We validated this finding by performing a case review of patients treated for schizophrenia and rhabdomyolysis at the Medical Center of the University of California, San Francisco (UCSF), thereby demonstrating the reproducibility of the identified association between schizophrenia and rhabdomyolysis. Multifactorial origins of rhabdomyolysis, such as adverse effects of drugs, catatonic stupor, neuroleptic malignant syndrome (NMS), and trauma^12–14^, have been reported. Our analytics highlighted an unexpected and idiopathic risk of rhabdomyolysis in schizophrenia patients.

Together, these findings demonstrate the power of using multi-level medical records, including public disease registries in combination with detailed electronic medical records, to discover novel disease associations, explore their etiologies, and inform clinical practice.

## Results

### Data set and overview of analytics

**Fig. 1:**
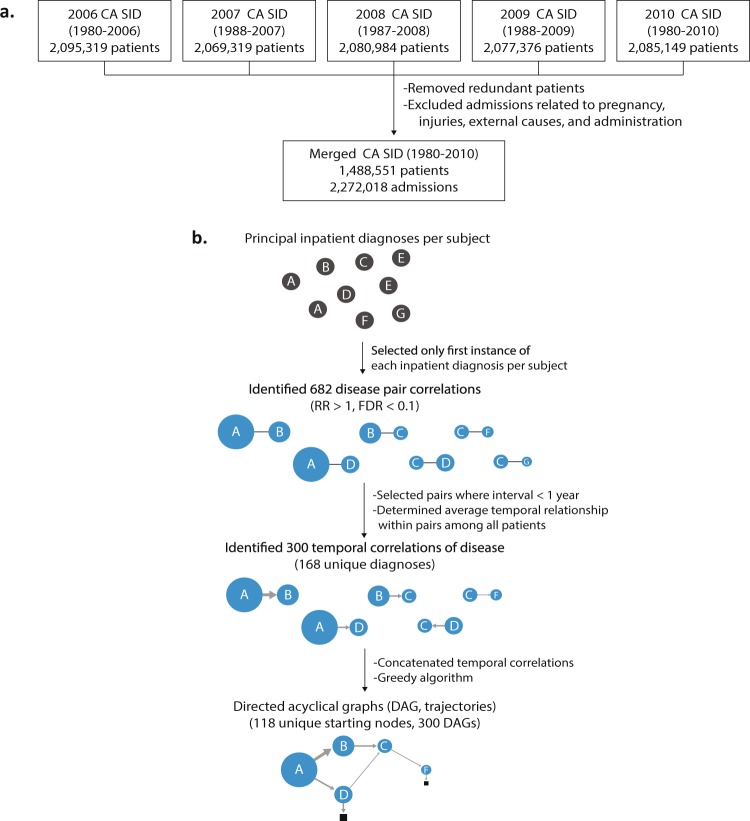
Overview of the main study data and analytics. (**a**) Overview of the data set build and analytics. We prepared the study data set by combining five editions of the California State Inpatient Database (CA SID) sets, which were released annually (2006–2010). In summary, we removed admissions for which primary diagnoses were not related to disease (pregnancy, injury, external causes, administration). The merged CA SID covers longitudinal records (1980–2010) for >1.4 million patients without data redundancy. (**b**) Construction of DAGs. The principal inpatient diagnoses for patients were selected and 682 significant disease association pairs (RA > 1, FDR < 0.1) were identified. The average temporal correlation was determined within pairs and then concatenated by using a greedy algorithm to create DAGs (i.e., trajectories) with 117 starting nodes.

As a main study set, we used the five editions of the annually released HCUP datasets from the CA SID, which contains inpatient billing records organized by the Agency for Healthcare Research and Quality (AHRQ) (https://www.hcup-us.ahrq.gov), including *International Classification of Diseases*, *Ninth Revision*, Clinical Modification (ICD-9-CM) diagnosis codes and death outcomes in hospitals, collected from over 350 community hospitals in California. The CA SID uses unique patient identifiers for each edition; thus, discharge data within the years is kept together for each patient within each edition, such as between 1980 and 2010 in the 2010 edition, but cannot be linked across previous editions. Therefore, while each edition of CA SID covers the longitudinal history of inpatient records for each patient across over 20 years (Fig. 1a), the patient record cannot be mapped between different editions of CA SID. To prevent recounting the same patients, we started with the 2010 data edition and then added record data from previous editions only for patients that were deceased during that year (and thus, could not have been admitted again in the 2010 edition) (see Methods and Fig. 1a, Supplementary Fig. S1A). The patients may have been treated for multiple illnesses during a hospitalization, both acute and chronic, so the first assigned diagnosis was used as the principal diagnosis for each admission. Given that we were interested in tracing the relationships of diseases, we filtered out ICD codes for non-disease conditions, which we defined as diagnosis chapters from ICD-9-CM, referring to injury, pregnancy, external causes, and healthcare-related administration codes. The merged data set covered 2,272,018 hospitalizations for 1,488,551 individuals with 290,253 death outcomes. The mean age of patients in the merged CA SID was 63.77 ± 19.58 years. (Supplementary Table S1).

As we were interested in temporal relationships between disease diagnoses within individuals, we simplified the timeline of each patient to the first instance of each primary diagnosis, such that readmissions for the same disease were removed. All diagnosis codes were reported using the ICD-9-CM and rounded to the three-digit code level^15^ to minimize overlap and subclassification of diagnoses (see Methods). Based on the intervals between the first and last admission dates for each individual, most of the merged CA SID data included diagnostic timelines for each patient of over three years (the median interval between the first and last admission dates per patient was 40.58 ± 2.5 months, see Supplementary Fig. S1C). The longest duration between the first and most recent admission dates in a patient was 26 years. We then identified significant disease associations by establishing the relative association (RA) of diagnoses co-occurring within one year in a patient^4,6^ using a binomial test, and compensating for multiple hypothesis testing using a false discovery rate (FDR) < 0.1 (see Methods)^7^. In short, RA is a ratio of the actual probability of co-diagnoses of two diseases over the random expectation of co-occurrences. When RA is greater than 1, it indicates the observed disease co-occurrence is higher than expected by chance (the details are shown in the Methods). We chose an interval of one year because the majority of new admissions of patients occurred within the same year, and we assumed admissions separated by very long-term intervals were less likely to be related.

Of the 691 three-digit level ICD-9-CM diagnosis codes used in the merged data set, 168 disease diagnoses were associated with at least one of the other 167 diseases, with a total of 300 temporally aligned disease pairs at FDR < 0.1. We determined the average time direction within each pair to determine the temporal relationship between diagnoses. Given that our data (CA SID) represent a statewide inpatient data set, the initial date of disease diagnosis is based on the date of admission for a disease. By using a greedy search algorithm^7^ (i.e., traced temporal relationships between diagnoses with the largest number of patients), we then concatenated these 300 temporal parings to create larger directed acyclic graphs (DAGs). By iterating over each starting disease compiled DAG, we retrospectively mapped 300 common trajectories from initial hospitalization through intermediate hospitalizations until death, if applicable (Fig. 1b). The 300 presented DAGs have 118 different diseases as nodes for the first diagnosis. The associated admissions cover 311,309 patients, who had 175,556 recorded readmissions for distinct diseases that are statistically correlated and that are presented as edges between disease diagnosis nodes. Of the traced patients, 34% (59,794) reached fatal outcomes as hospital inpatients.

### DAGs depict temporal disease associations and mortality

In total, we identified 300 major trajectories (i.e., DAGs) consisting of nodes for disease diagnoses, and edges for subsequent readmission for statistically associated disease diagnoses. Of the 300 trajectories, the longest trajectory had four readmission steps for correlated diseases from the initial presentation of disease diagnosis. In 257 trajectories, the latest steps of readmissions are associated with fatal outcomes, i.e., deaths in the care setting. In 43 cases, the traced patients were either still alive or there were no death outcomes at the final disease diagnosed (Supplementary Fig. S1D). While the dataset is too comprehensive to review all our findings in detail (see Appendix Movie S1. Dynamic presentation of the traced diagnosis trajectories at scale; https://www.youtube.com/watch?v=jJMds31-e2g), we highlight a few selected trajectories (i.e., DAGs).

**Fig. 2:**
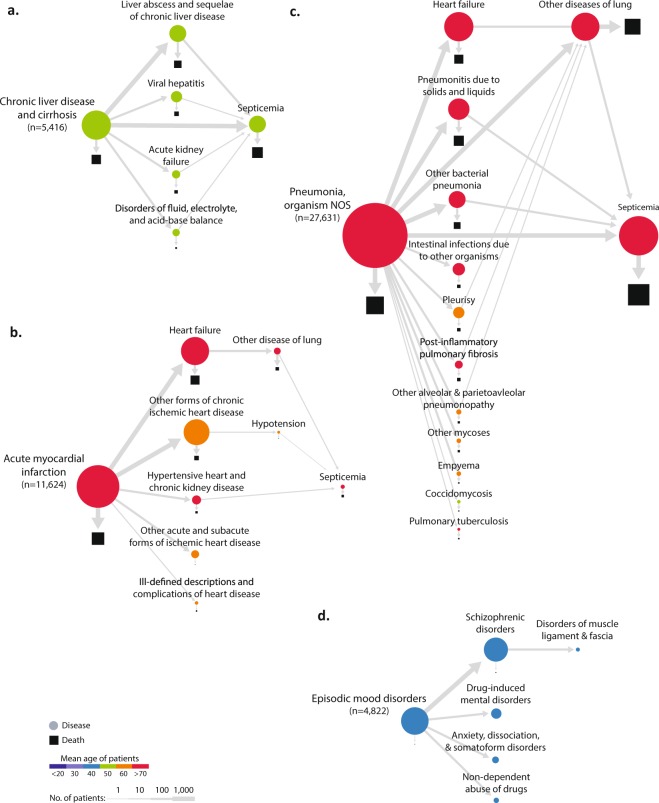
Selected disease trajectories (DAGs). Selected DAGs (117 in total) showing trajectories for one-year intervals between primary diagnosis codes for California inpatient admissions. Areas of shapes are directly proportional to the number of patients, where circles represent primary diagnoses and squares represent deaths. Nodes are colored by mean age of patients. The thickness of edges is determined by the number of patients traveling along the edge (see Legend). (**a**) DAG of “chronic liver disease and cirrhosis.” Prominent nodes include “liver abscess and sequelae of chronic liver disease,” “septicemia,” and “death.” (**b**) DAG of “acute myocardial infarction.” The majority of readmissions were for “heart failure” and “other forms of chronic ischemic heart disease,” and “acute myocardial infarction” and “heart failure” were the nodes most associated with death. Note that some of these second diagnoses occur in a larger population of patients beyond those coming in with acute myocardial infarction, and this can lower their mean age of incidence. (**c**) DAG of “pneumonia, organism NOS.” Common readmissions were for “septicemia,” “heart failure,” “other disease of lung,” “pneumonitis due to solids and liquids,” and “other bacterial pneumonia.” These diagnoses were all strongly associated with death. (**d**) DAG of “episodic mood disorders,” which includes diagnosis codes for uncharacterized mental disorders. The majority of hospitalizations were readmitted for diagnoses of “schizophrenia” and a significant proportion of these individuals were later hospitalized for “disorders of muscle ligament and fascia;” 93% of the admissions for “disorders of muscle ligament and fascia” were more specifically coded for rhabdomyolysis (ICD-9-CM code = 728.88).

We focused on three DAGs within the network that represent the disease trajectories leading to the most hospital deaths in California (Fig. 2). Among patients in their fifties, most hospital deaths (2,237 deaths) occurred within the DAG associated with a “chronic liver disease and cirrhosis,” which was the initial presentation of disease in the DAG for 5,416 patients. A significant portion (up to 35%) experienced infection complications (liver abscess and sequelae of chronic liver disease)^16^ and then sepsis, with most deaths associated with these ICD diagnoses (Fig. 2a). We also recapitulated a well-known phenomenon that acute myocardial infarction commonly leads to heart failure^17^ and that these steps of diseases have high mortality (19%; 2,245 deaths among 11,624 of the traced acute myocardial infarction patients) (Fig. 2b). Thus, identified diagnosis patterns within DAGs appeared to be reliable and represent currently understood phenomena.

We also present the DAG for pneumonia, which disproportionately affects older individuals (mean age in the 70 s). This DAG demonstrated a network with both high morbidity and mortality. Associated subsequent diagnoses included heart failure and subsequent lung disease^3^, aspiration pneumonia (pneumonitis due to solids and liquids), and sepsis (Fig. 2c). Indeed, sepsis was a common dead-end node in many DAGs, which reinforces previous studies demonstrating the high prevalence of sepsis in the USA^18^. Together, these findings allow common disease paths to be traced and enables diseases of high mortality in clinics to be identified in a systematic fashion. In addition to recapitulating identified disease trajectories, greater detail of 300 trajectories (i.e., DAGs) are presented in the appendix data of traced diagnosis trajectories (https://github.com/hypaik/HCUPSIDCA_trajectory_tracking). Moreover, details of all modeled trajectories, which are described in Appendix Data S1, might be helpful to understand the heterogeneity of patients for clinical care (https://github.com/hypaik/HCUPSIDCA_trajectory_tracking). For example, osteomyelitis periostitis patients (i.e., inflammation of bone, ICD-9 code: 730) were stratified based on the correlated previous diagnoses, cellulitis, and diabetes.

### Schizophrenia is associated with subsequent hospitalization for rhabdomyolysis

**Fig. 3:**
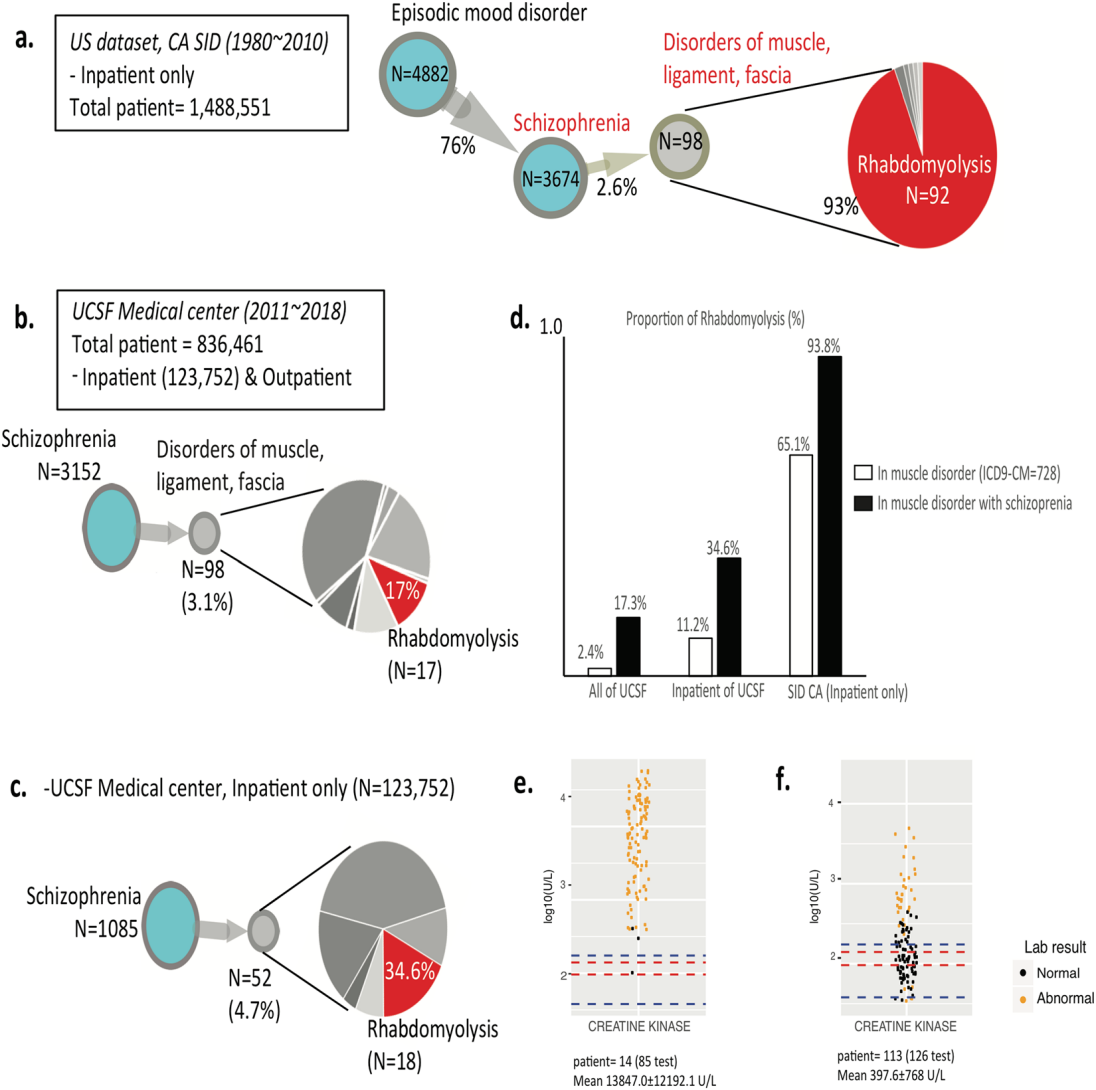
The identified novel association between schizophrenia and rhabdomyolysis. Our scaled analysis of digitalized medical records from millions of patients reveals a novel association between schizophrenia and rhabdomyolysis. (**a**) DAG of schizophrenia and disorders of muscle, ligament, and fascia enriched with rhabdomyolysis based on the data of CA SID. (**b**) DAG of schizophrenia and disorders of muscle, ligament, and fascia enriched with rhabdomyolysis based on the EHRs of the UCSF Medical Center. (**c**) The identical DAG of (**b**) using the subset of the EHRs of the UCSF Medical Center consisting of inpatient records. (**d**) The proportion of the diagnoses of rhabdomyolysis in schizophrenia across different data sets. Black bars indicate the fraction of rhabdomyolysis among the muscle disease (ICD-9-CM code = 728) in schizophrenia. White bars represent the rate of rhabdomyolysis in muscle disease-diagnosed patients. (**e**) Lab test evidence for the diagnoses of rhabdomyolysis in (B). The levels of creatine kinase in patients who were diagnosed with rhabdomyolysis after schizophrenia are extremely high. (**f**) The level of creatine kinase in patients who were diagnosed with schizophrenia, but not diagnosed with rhabdomyolysis in (**b**). Because those patients already suffer with illness, the creatine kinase levels are mildly increased but these values are far from those of (**f**). The blue and red lines are the reference range for normal levels of creatine kinase (38–174 units/L [reference for male, blue line]; 96–140 units/L [reference for female, red line]). The colors of dots indicate normality or abnormality marks assigned by healthcare providers (black for normal and orange for abnormal tags).

We also found a surprising relationship between schizophrenia and muscle disorders (Fig. 2d). In this DAG, 4,822 patients were first diagnosed with episodic mood disorders, which are often used in initial diagnoses for psychiatric diseases while medical providers collect collateral data to make a more specific clinical diagnosis. As expected, 76% (3,674) were subsequently readmitted with schizophrenia (schizophrenic disorders). While diagnoses for psychotic disorders exist on a continuum of clinical symptoms, the fifth edition of the *Diagnostic and Statistical Manual of Mental Disorders* (DSM-V) requires either two outpatient evaluations or one inpatient hospitalization to make a diagnosis for schizophrenia. Therefore, we felt confident in this categorization. Interestingly, 98 of the 3,674 patients (2.6%) with schizophrenia were readmitted within one year with muscle disorders. Of these 98 patients with disorders of muscle, ligament, and fascia, 92 (2.5% of 3,674 schizophrenia patients) were more specifically coded as having rhabdomyolysis, which is usually a rare disease of muscle breakdown that can lead to kidney failure or even death (relative risk of rhabdomyolysis [RR] = 2.21 [1.80–2.71, confidence interval (CI) = 0.95] *P*-value of RR 9.54E-15, RA = 1.5, mean interval 114.9 ± 84.3 days, FDR of RA and temporal order = 8.76E-02) (Fig. 3a). Although it has been historically very difficult to study the epidemiology of rhabdomyolysis, an incidence of lower than 0.0001 (26,000/year among the 325 million US population, or an incidence of 8E-05 per year) has been estimated^19^. Thus, a rate of 2.5% in a specific patient population (schizophrenia) was peculiarly high (313-fold higher than a population-wide incidence of rhabdomyolysis; 0.026 is 325 times greater than 8E-05) (*P*-value of enrichment using hypergeometric test 7.20E-03). There has been one case report describing the use of second-generation antipsychotics, such as aripiprazole^20^ (a schizophrenia drug for adolescent patients^21^), being associated with rhabdomyolysis in a schizophrenia patient. Regarding the rare and immediate adverse effect of aripiprazole, the association with schizophrenia is not a widely known disease association (calculated mean interval between schizophrenia and rhabdomyolysis = 114.9 ± 84.3 days). Thus, we sought to verify and further characterize this novel relationship.

### Multi-level validation of patients with schizophrenia diagnosed with rhabdomyolysis

To better understand the potential etiology of rhabdomyolysis in schizophrenia patients, we searched for this relationship using deidentified electronic health records (EHRs) from the UCSF Medical Center. While UCSF is one of the contributors to the CA SID, the UCSF EHR data we used consisted of inpatient and outpatient encounters from 836,461 patients between 2011–2018 (inpatients = 123,752 patients), whereas the merged CA SID we used consisted of five editions of CA SIDs covering inpatients between 1980 and 2010. In addition, the CA SID data covers nearly 69 times the number of discharges compared with UCSF. Thus, we believe the data in the UCSF and CA SID do not overlap, and if they do, the overlap is minimal; however given that both datasets are deidentified, we were not able to verify this. We began by using UCSF’s deidentified EHR Clinical Data Warehouse, which contains structured data including diagnosis codes, lab values, vital signs, procedure codes, and administered medications, without any clinical text, images, or actual encounter dates or patient identifiers.

Similar to CA SID data, we found the proportion of UCSF patients with schizophrenia who were treated for “disorders of muscle ligaments and fascia” to be statistically higher than expected (RA = 1.1, mean interval = 296.7 ± 311.1 days, FDR < 0.1). When we tracked these data in a time-ordered manner, 98 of 3,152 schizophrenia patients (3.1%) were hospitalized or visited the medical center for muscle disorder after the schizophrenia, and 17 of those 98 (17.3%) were enriched with rhabdomyolysis (*P*-value of hypergeometric test 9.9E-22) (Fig. 3b). In UCSF, the number of rhabdomyolysis patients was 541 among 836,461 patients (0.06%). Thus, in the UCSF Medical Center, the diagnosis of rhabdomyolysis is also peculiarly high among schizophrenia-diagnosed patients (0.53%, 17 of 3152, RR = 18.3 [13.4–25.0, CI = 0.95] *P*-value of RR = 2.2E-16). A similar pattern was captured among 1,085 schizophrenia inpatients of the UCSF Medical Center (*P*-value of enrichment using hypergeometric test 1.51E-11) (Fig. 3c) (1.65%, 18 of 1085, RR = 9.61 [6.8–13.4, CI = 0.95] *P*-value of RR = 1.1E-15). Although the Columbia Open Health data does not provide a way to query using temporal order, the identical association between schizophrenia and rhabdomyolysis was displayed in their data resource as a matched consistency (log ratio of observed co-occurrences than random = 3.01)^22^.

In addition, the enrichment patterns of rhabdomyolysis diagnoses among the muscle disorders in schizophrenia are repeated across the data of UCSF EHRs and CA SID (Fig. 3d). Among 22,374 muscle disease patients in our UCSF data set (ICD-9-CM code ‘728’ for disorders of muscle, ligament, and fascia), only 541 (2.4%; shown as a white bar in Fig. 3D) had rhabdomyolysis (ICD-9-CM code ‘728.88’). However, the diagnosis fraction of rhabdomyolysis was sevenfold higher among the overall muscle diseases in schizophrenia patients in UCSF (17.3%, 17 of 98 muscle disease patients with schizophrenia, shown as a black bar). The black bars in Fig. 3D represent the proportions of patients that were diagnosed with rhabdomyolysis among patients diagnosed with both muscle disorder and schizophrenia. These proportions were larger than the fraction of rhabdomyolysis in overall muscle disorder in UCSF EHRs and CA SID, which are indicated by the white bars.

We also examined whether rhabdomyolysis occurred following schizophrenia due to the adverse effect of aripiprazole, a second-generation antipsychotic^20^; however, we could find no evidence that any of these schizophrenia inpatients were administrated aripiprazole within the UCSF Medical Center. Other antipsychotic drugs, including haloperidol and clozapine, have less clear association with rhabdomyolysis (*P*-value of odds ratio > 0.05); we believe the association between schizophrenia and rhabdomyolysis in UCSF is, thus, independent of adverse drug effects.

The diagnosis codes for rhabdomyolysis, often assigned for billing purposes, were also validated based on clinical evidence (lab values). The levels of creatine kinase (CK) for the diagnostic confirmation of rhabdomyolysis greatly exceeded the normal reference (Fig. 3e) (normal reference of CK = 38–174 U/L (reference for male, blue line in Fig. 3e); 96–140 U/L (reference for female, red line in Fig. 3e)) in schizophrenia–rhabdomyolysis patients (mean = 13847.0 ± 12192.1 U/L), which allowed a high confidence in the correct diagnoses of rhabdomyolysis occurrence in patients with schizophrenia (14 of 17 patients in Fig. 3b).

Although mild increases of CK levels are known to be relevant to muscle pain, metabolic abnormality, and drug overdose, only rhabdomyolysis is associated with extremely high CK values(10,000–200,000 U/L), due to the severe muscle disruption, which later can lead to renal failure^23^. To confirm a diagnosis of the condition, physicians have used the CK level test for patients who having relevant symptoms. In our previous work, we confirmed that the examination of CK levels are rarely ordered in among healthy individuals^24^, which suggests that CK levels are likely examined only for individuals having some prior probability of a muscle relevant symptom. As we expected, in the compared control group, slight elevations in CK levels among schizophrenia patients without rhabdomyolysis are observed in the EHR data of UCSF (Fig. 3f, mean = 397.6 ± 768 U/L). Indeed, simply requesting a CK level itself is suggestive that there is an elevation. The control patients at UCSF are probably enriched with a skewed higher distribution of CK levels. However, mechanistic support for the proper diagnosis of rhabdomyolysis in these patients is still clear from the strikingly high CK level.

We then proceeded to conduct detailed case reviews, after obtaining approval from the UCSF Institutional Review Board (approval number 17-22258). We gathered the data from 29 cases in which rhabdomyolysis was diagnosed after schizophrenia. A case review of physicians’ notes (conducted by professional medical providers, including help from psychiatry) for patients with schizophrenia treated for rhabdomyolysis at UCSF between 2011 and 2018 (*n* = 29) revealed that 37% of these cases (*n* = 11) involved illicit drug ingestions, and 13% (*n* = 4) were also from fractures/falls. Other muscle symptoms in schizophrenia that can be relevant to rhabdomyolysis, such as neuroleptic malignant syndrome (NMS), catatonic seizure and spasm, were not detected from the healthcare records including physicians’ notes on these subsets of schizophrenia patients who were diagnosed with rhabdomyolysis at UCSF^25^. However, over half were of idiopathic origin (*n* = 18, 62%), and had no clinically known cause, including any known direct adverse effect of a medication. This suggests that rhabdomyolysis could be an unexpected comorbidity for schizophrenia patients. Due the low background incidence of rhabdomyolysis, it is possible that even more schizophrenia patients have rhabdomyolysis than recognized. If this association continues to be validated, psychiatrists could be counselled to consider a higher probability for rhabdomyolysis in schizophrenia patients.

## Discussion

From the population-scale analysis of digital health records, the unexpected propensity of rhabdomyolysis to occur in patients with schizophrenia (0.5–2.5%) was consistently detected compared with random and sporadic chances across the two data sets composed of tens of millions of individuals (CA SID and UCSF EHRs, RRs 2.21–18.35). Known possible risk factors for rhabdomyolysis, such as adverse effects of medication and neuroleptic malignant syndrome (NMS), were not reported in the majority of rhabdomyolysis cases following schizophrenia. Thus, our state-level scaled analysis of digitalized medical records from millions of patients reveals a novel association between schizophrenia and rhabdomyolysis.

This study included an extensive temporal analysis of disease occurrences across the discharge records of 10.4 million patients in Californian hospitals. In total, we identified 300 serial readmissions for diverse disease diagnoses beginning with 118 disease nodes in 311,309 patients. Throughout 175,556 succeeding admissions with temporally correlated diagnosis from previous admissions and a relevant diagnosis, our approach presents time-aligned patterns of readmission and consistently modeled clinical associations, such as heart failure following acute myocardial infarction, and sepsis in cancer patients^26^. Details of all modeled trajectories are described in Appendix Data S1 (and at https://github.com/hypaik/HCUPSIDCA_trajectory_tracking).

Interestingly, from the modeled disease trajectories, we repeatedly identified a novel association of diseases, including rhabdomyolysis following schizophrenia, from the independent data set including UCSF EHR. By combining multilevel validation and case reviews of these patients with professional medical providers, an unexpected risk of rhabdomyolysis in schizophrenia was revealed. This finding constitutes direct evidence for the value of digital health records for patient care in influencing clinical practice in a systematic fashion, such as identifying the risk of further disease.

It is known that patients with schizophrenia and other serious psychoses are underdiagnosed and undertreated for comorbid illnesses, such as hypertension and diabetes^27^. Thus, given the absence of the observational data recapitulation and time-ordered presentation, the association with rhabdomyolysis would have been overlooked. Potential etiologies include drug associations because patients with schizophrenia are often treated with antipsychotic medications, and there is a known relationship between second-generation antipsychotics causing NMS, a disease of catatonia and muscle spasm that can lead to muscle breakdown. There have also been rare case reports of second-generation antipsychotic use in patients with schizophrenia causing rhabdomyolysis in the absence of NMS. Patients with schizophrenia are more vulnerable to socioeconomic stressors, such as illicit substance use and homelessness, which are all independent risk factors for rhabdomyolysis. Although these explanations are still more likely, there could also be biological explanations. There are known brain disorders, including schizophrenia, that share genetic etiologies with diverse diseases^28^. Indeed, over 108 genetic loci are associated with schizophrenia^29^. It is possible that an underlying genetic predisposition can affect both neurons and muscle cells. For example, *AHI1* is associated with both schizophrenia and metabolic abnormalities of muscles^30,31^ and perhaps could contribute to these two disease predispositions.

Several limitations of the study should be noted. Other diverse and overlooked clinical and patient features are invisible in our data, such as neglected symptoms including mild muscle spasm, and unreported behaviors relevant to rhabdomyolysis after the discharge of patients. For example, deaths occurring at home are truncated in our data set^32^. In further studies, finer acquisition of EHRs for the computational approaches, such as natural language processing of the chart records, will help in the care of schizophrenia patients and facilitate risk assessment for rhabdomyolysis. Moreover, our trajectory model traced re-admissions within one-year. More longitudinal re-admission pattern would be helpful for long-term care of patients in further studies. However, based on our findings, the awareness of this rare but critical risk (i.e., rhabdomyolysis) in schizophrenia patients may be helpful for providing adequate clinical practice. Further studies on the shared mechanistic or biological etiologies between schizophrenia and rhabdomyolysis should be conducted.

Two key messages can be deduced from this study. First, the analysis of health records can aid in advancing clinical knowledge for patient care. While most physicians are eager to comply with the latest evidence guiding clinical practice, scientific inquiries have been slow to uptake the rapidly expanding amount of data that are available from clinics. By using advanced data-mining analytics, we demonstrate how large-scale digital healthcare data can be used to facilitate the generation of medical knowledge. Second, the validation of the novel association between schizophrenia and rhabdomyolysis from the large-scale analytics depends mainly on the multilevel evidence of health records including finer validation of EHRs using lab test results and chart review with clinicians. Thus, for the specific contribution of big-data analytics and to effectively draw beneficial inference from clinical practice, communication with data scientists and physicians who have insight from routine clinical practice is indispensable. Although disease registries including CA SID are useful resources for observational studies, the use of a diverse range of evidence from clinical records, such as EHRs, are essential for novel findings. In this report, we have focused on a single example of our large-scale analysis (rhabdomyolysis after schizophrenia). To disseminate our findings, all our results are available on an interactive website where the mapped associations of diseases can be explored via dynamic visualization. Appendix Movie S1 (Dynamic presentation of the traced diagnosis trajectories at scale; https://www.youtube.com/watch?v=jJMds31-e2g) and Appendix Data S1 (All traced diagnosis trajectories; https://github.com/hypaik/HCUPSIDCA_trajectory_tracking) presents a preview of the website (http://52.89.56.137:3000/#/intro, optimized for Chrome).

By tracking millions of healthcare records, this study has revealed a novel and consistent propensity for schizophrenia patients to develop rhabdomyolysis. Digital health data are a promising resource that can be used to create observational evidence for clinical questions, such as readmission for relevant diseases, and to identify novel risk of disease patients, which can influence clinical practice. Together, these findings demonstrate the power of using new analytic methods on large disease registries to understand disease and mortality risk factors, particularly for rare but preventable diseases or outcomes.

## Methods

### California administrative healthcare records dataset

We used the HCUP CA SID from the AHRQ. This database contains deidentified admission and discharge billing information from over 350 community hospitals in California, including nonfederal, general, specialty, and academic medical centers. The CA SID excludes non-community hospitals, such as federal hospitals (e.g., Veterans Affairs), long-term care hospitals, and clinical units within institutions (e.g., prisons).

We utilized five of the most recent annual builds of CA SID, which included 364 hospitals in edition 2006 (longitudinal inpatient records between 1980–2006), 360 hospitals in edition 2007 (records between 1988–2007), 361 hospitals in edition 2008 (records between 1987–2008), 354 hospitals in edition 2009 (records between 1988–2009), and 354 hospitals in edition 2010 (records between 1980–2010). We merged these five CA SID editions, each of which consists of over 20 years of longitudinal records for about 2 million patients. Thus, the merged CA SID covers inpatient records between 1980 and 2010. While each edition of CA SID used unique identifiers for every individual, allowing us to trace patients temporally across hospitals in a longitudinal manner, these identifiers were not consistent across versions, preventing meta-mapping of the patient identifiers between editions. To prevent data redundancy in the merged CA SID data set, we used records of only deceased individuals and their hospitalization records in the 2006–2009 editions, and merged these with the most recent 2010 edition.

All diagnosis codes were reported using the ICD-9-CM and rounded to the three-digit code level^15^ to minimize overlap and subclassification of diagnoses. For example, the three-digit ICD-9-CM code for vascular dementia (F01) involves subtypes consisting of dementia with or without behavioral disturbances (F01.50 or F01.51); therefore, we used three-digit ICD-9-CM codes to avoid diagnosis subclassifications for vascular dementia.

### Temporal disease diagnoses correlations

We used the first charted diagnosis as a primary diagnosis code to determine the main reason for each patient hospitalization. To determine the temporal association of admission diagnoses correlations, we first calculated the RA measurement of all disease pairs (*Disease i – Disease j*) that occurred within one year for each patient^6^. Here, we used diagnoses for the patients based on the assigned three-digit ICD-9-CM code. RA measures relative ratio of disease co-occurrences over incidences of compared disease pair. The starting point of our RA measure is the database (CA SID) containing the diagnoses that *C*_*ij*_ led to all hospitalization (*N*) of each disease identified by an ICD-9-CM code. We denote the incidence of disease *i* with *Ii*, and the number of patients who were diagnosed with diseases *i* and *j* with *C*_*ij*_. The co-occurrence value, between diseases can be quantified as RA = *C*_*ij*_/*C*_ij_^*^, where *C*_ij_^*^ = *I*_*i*_ • *I*_*j*_/*N* is the random expectation value of *C*_*ij*_. The statistical significance of disease comorbidities were determined by using the binomial test based on a previous study^7^. We selected pairs where RA was greater than one, which indicated that the co-occurrence of the two diseases was higher than expected by chance. Then, by modifying a previous method, we determined the likelihood within a pair of diseases for one disease to occur before the other (${\delta }_{i\to j}$ for *disease i → disease j*) by using the dates of admissions associated with the two diseases in each patient^4^. To calculate ${\delta }_{i\to j}$, we compared diagnoses dated for each patient. We defined ${d}_{i\to j}^{p}\in \{-1,1\}$ as 1 if disease *i* was diagnosed before disease *j* in patient *p* and −1 otherwise. Multiple rediagnoses or rehospitalizations for the same diseases in the same patient were ignored and only the initial date of admission for a disease was used as a date of diagnosis for the disease. In addition, we only counted ${d}_{i\to j}^{p}$ when the time between two admission dates was less than one year to filter out cases in which the diagnosis happened over a very long-term duration. A value of ${d}_{i\to j}^{p}$ > 0 indicates the following: an initial admission for disease *i* occurred before the first admission for disease *j* in a patient *p* within one year. Then, the value of ${\delta }_{i\to j}$ was determined by the mean value of ${d}_{i\to j}^{p}$ among the set of patients who were diagnosed with diseases *i* and *j* within one year. Thus, a value of ${\delta }_{i\to j}$ > 0 indicates that over half of the admissions for disease *i* occurred before the admissions for disease *j* by one year among the patients who were diagnosed with both diseases. The statistical significance of disease pair co-occurrences (RA) and temporal directionality of diseases (${\delta }_{i\to j}$) were determined by using a binomial test (Benjamini–Hochberg FDR < 0.1)^7^. Finally, we used pairs of correlated diseases with time directionality whose mathematical relationships were statistically significant (RA > 1, FDR < 0.1; ${\delta }_{i\to j}$ ≠ 0, FDR < 0.1) for further analysis. We use the term ‘temporal disease correlation’ to describe this relationship.

### Disease trajectories (DAGs)

We joined multiple temporal disease correlations by concatenating temporal disease correlations into three or more steps of overall disease occurrences among patients (i.e., Disease 1 → 2 and Disease 2 → 3 to form Disease 1 → 2 → 3)^7^. Because our graph model only concedes a directional pair of temporal disease correlation, the result of concatenation is a directed acyclic graph (DAG), which means that the model presents a serial pattern of disease diagnoses by time without a loop (i.e., relapse of the same disease diagnoses again). A greedy approach was used to find subsequent steps in disease paths. Disease pairs were sorted in descending order according to patient counts. Pairs with overlapping diagnoses were found starting from the top of the list, and the number of patients following the full trajectory to death was counted. The process was stopped when a trajectory had no patients following it.

### Data validation and case review using USCF EHRs

We utilized EHRs from UCSF collected using the Epic system (Verona, WI) between 2011 and 2018, which includes inpatient and outpatient records for 836,461 unique individuals. The records were deidentified and contained no direct patient identifiers, as defined in the Health Insurance Portability and Accountability Act (HIPAA). For the manual case review of schizophrenia and rhabdomyolysis patients, the analysis protocols were reviewed and approved by the UCSF Institutional Review Board (approval number 17-22258).

## Supplementary information

Supplemental Table S1.

Supplementary Figure S1.

## Data Availability

Inpatient records of over 10.4 million patients from California hospitals are available via material transfer agreement with the HCUP of the AHRQ (https://www.ahrq.gov/research/data/hcup/). The de-identified UCSF EHRs covering 836,461 patients who were diagnosed at UCSF between 2011–2018 are only available from UCSF via inter-institutional agreement, to protect the personal information of individuals.

## Code Availability

The following software and source codes were used for the presented analysis and described in the main text:

1. MySQL v14.12 was used for analysis of HCUP SIDCA data. https://www.mysql.com/downloads/

2. An in-house developed Java script was used to trace the diagnosis trajectories of over 10.2 million patients. The source codes are available via GitHub repository for academic researches.

https://github.com/hypaik/HCUPSIDCA_trajectory_tracking

3. R package version 3.2, Java v1.8.0 and Python v2.7 were used for the presented statistical analysis.

https://www.r-project.org/

https://www.oracle.com/technetwork/java/javase/downloads/jdk8-downloads-2133151.html

https://www.python.org/download/releases/2.7/

4. Cytoscape, an open source platform, was used for the network analysis of disease trajectories and figure preparations. https://cytoscape.org/

5. WebGL was used for the dynamic visualization of the presented disease trajectories.

https://developer.mozilla.org/en-US/docs/Web/API/WebGL_API

6. All the traced disease trajectories for over 10.4 million patients in California, US was presented via our web resource.

http://52.89.56.137:3000/#/intro (Optimized only for Chrome)

7. We also have a website and video explaining these disease trajectories. (Appendix Movie S1. Dynamic presentation of the traced diagnosis trajectories at scale)

https://www.youtube.com/watch?v=jJMds31-e2g

8. Appendix Data S1. All of traced diagnosis trajectories. (https://github.com/hypaik/HCUPSIDCA_trajectory_tracking).
