# Supplementary material for: Tracing diagnosis trajectories over millions of patients reveal an unexpected risk in schizophrenia
Source: Sci Data. 2019 Oct 15;6:201. doi: 10.1038/s41597-019-0220-5 (PMC6794302; doi:10.1038/s41597-019-0220-5)
Supplement: Supplementary file 1 — Supplemental Table S1. [file 41597_2019_220_MOESM1_ESM.docx]

**Supplemental Table 1.** Data statistics of California State Inpatient Database (SID CA)^*^

| Features | No. recorded (in selected set)^3^ |
| --- | --- |
| No. of admissions  Data build in 2006^1^ (1980–2006)^2^  Data build in 2007^1^ (1988–2007)^2^  Data build in 2008^1^ (1987–2008)^2^  Data build in 2009^1^ (1988–2009)^2^  Data build in 2010^1^ (1980–2010)^2^ | 19,984,041 (**2,272,018)**  3,997,182 (128,516)  4,012,774 (126,768)  4,017,998 (124,632)  3,985,166 (121,661)  3,970,921 (1,770,441) |
| Grand total no. of patients  Data build in 2006  Data build in 2007  Data build in 2008  Data build in 2009  Data build in 2010 | 10,408,641 (**1,488,551)**  2,095,319 (68,237)  2,069,813 (66,611)  2,080,984 (66,048)  2,077,376 (63,691)  2,085,149 (1,223,964) |
| No. of unique diagnosis codes (ICD-9-CM)  No. of unique 3-digit diagnosis codes | 5,777  691 |
| Demographic features (in selected set)  Mean of age in the admission month  Gender  Outcomes of admission  Deaths  Survival | 63.77 (±19.58) years  M: 691,452 (46.4%), F: 780,230 (52.4%)  290,253 (19.5%)  1,334,635 |

^*^ Data resource of the Healthcare Cost and Utilization Project (HCUP) covering 97% of hospitals in the US, California.

^1^ Years of data set generations. Merged data set covers up to ~26.1 years of longitudinal events for a patient counted by administration month. For each inpatient event, up to 25 diagnosis codes were assigned. ^2^ Covered years of records by build year versions. ^3^ Excluded by diagnosis chapters for injury, symptom, childbirth, pregnancy, and healthcare service.
