# Supplementary material for: Tracing diagnosis trajectories over millions of patients reveal an unexpected risk in schizophrenia
Source: Sci Data. 2019 Oct 15;6:201. doi: 10.1038/s41597-019-0220-5 (PMC6794302; doi:10.1038/s41597-019-0220-5)
Supplement: Supplementary file 2 — Supplementary Figure S1. [file 41597_2019_220_MOESM2_ESM.pdf]

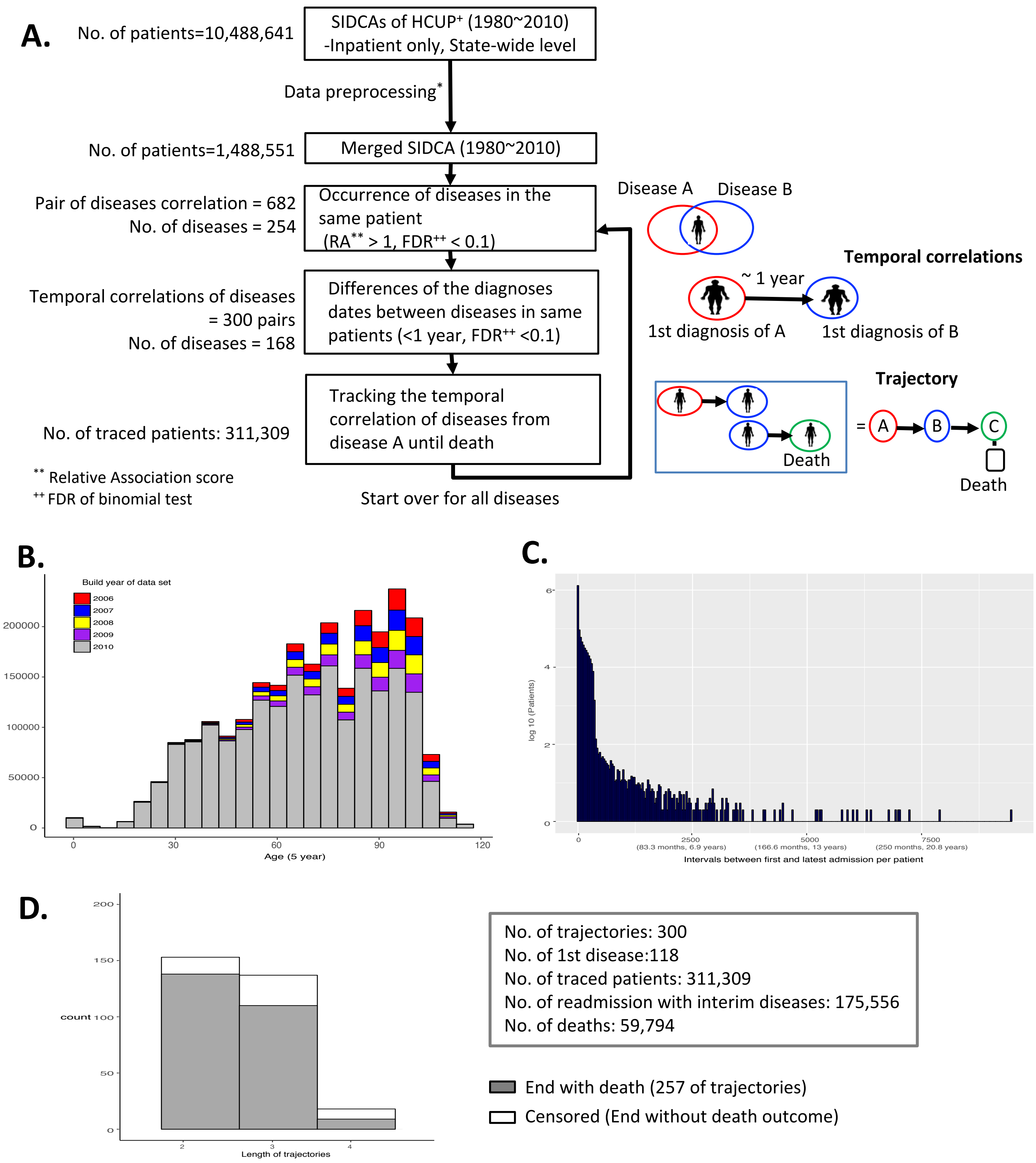

+ State Inpatient Database, California (SIDCA) of the Healthcare Cost and Utilization Project (HCUP).

* Excluded external injury, childbirth, pregnancy and health care service-related contact by diagnosis codes.

Supplementary figure S1. Overview of a main study data (SIDCA) and traced disease trajectories.
A. Over view of data filtering procedure. The detailed statistics of Merged SIDCA were presented in Supplemental Table 1. **B.** Data distribution of the Merged SIDCA. **C.** Time intervals between the first and the latest admission for each patient. **D.** Length distribution of traced disease trajectories.
